# Habitat generalists and specialists in microbial communities across a terrestrial-freshwater gradient

**DOI:** 10.1038/srep37719

**Published:** 2016-11-25

**Authors:** C. Monard, S. Gantner, S. Bertilsson, S. Hallin, J. Stenlid

**Affiliations:** 1 Department of Forest Mycology and Plant Pathology, Uppsala BioCenter, Swedish University of Agricultural Sciences, P.O. Box 7026, SE-75007 Uppsala, Sweden; 2 Department of Ecology and Genetics, Limnology and Science for Life Laboratory, Uppsala University, Norbyvägen 18D, SE-75236 Uppsala, Sweden

## Abstract

Observations of distributions of microorganisms and their differences in community composition across habitats provide evidence of biogeographical patterns. However, little is known about the processes controlling transfers across habitat gradients. By analysing the overall microbial community composition (bacteria, fungi, archaea) across a terrestrial-freshwater gradient, the aim of this study was to understand the spatial distribution patterns of populations and identify taxa capable of crossing biome borders. Barcoded 454 pyrosequencing of taxonomic gene markers was used to describe the microbial communities in adjacent soil, freshwater and sediment samples and study the role of biotic and spatial factors in shaping their composition. Few habitat generalists but a high number of specialists were detected indicating that microbial community composition was mainly regulated by species sorting and niche partitioning. Biotic interactions within microbial groups based on an association network underlined the importance of *Actinobacteria*, *Sordariomycetes*, *Agaricomycetes* and *Nitrososphaerales* in connecting among biomes. Even if dispersion seemed limited, the shore of the lake represented a transition area, allowing populations to cross the biome boundaries. In finding few broadly distributed populations, our study points to biome specialization within microbial communities with limited potential for dispersal and colonization of new habitats along the terrestrial-freshwater continuum.

Microorganisms, comprising much of the biodiversity on Earth, play major roles in functions and processes in a wide range of ecosystems such as soils or freshwaters^1^. Bacterial communities are central to most biogeochemical cycles in both soils and aquatic ecosystems^2^,^3^,^4^. Fungi are ubiquitous and diverse. They play a dominant role in decomposition and nutrient cycling in soil through their saprotrophic activities, take part in mycorrhizal associations in soil and can be abundant in freshwaters^3^,^5^,^6^,^7^,^8^,^9^,^10^. Archaea are also widespread and phylogenetically diverse in terrestrial and freshwater ecosystems, the latter being considered as the largest reservoirs of archaeal genetic diversity^11^,^12^,^13^. Archaea are actively participating in ecosystem processes such as methanogenesis and nitrogen cycle^14^. Whereas ecosystem functioning can be affected by the identity and relative abundance of microbial taxa^15^,^16^,^17^, an understanding of the local distribution of microorganisms within the microbial metacommunity is needed to improve predictions of ecosystem responses to global change.

Distributions and co-occurrence patterns of microorganisms as well as differences in community composition across habitats (beta diversity) provide evidence of biogeographical patterns for microorganisms^18^,^19^,^20^,^21^. Both deterministic and stochastic processes have been used to explain microbial community assembly^22^,^23^. Deterministic processes assume that community composition is driven by environmental factors leading to species sorting^24^. Species interactions and competition may further affect the composition of microbial communities and drive their distribution patterns^25^,^26^,^27^,^28^. Stochastic processes on the other hand control community composition by dispersal limitation, mass effect and random birth/death events^29^. Microbial community composition seems to be governed by both deterministic and stochastic processes^30^. However, depending on the habitat, the spatial scale and the community considered the relative influence of these processes will differ^23^,^31^. It has been shown that habitat generalists, showing broad environmental tolerances, and habitat specialists, having more restricted habitat ranges, are assembled by different mechanisms leading to complex metacommunity dynamics^32^,^33^.

Most studies on distribution of microorganisms, as well as structure and turn-over of microbial communities focus on one biome and little is known about the processes mediating and controlling transfers across a landscape gradient involving terrestrial and aquatic biomes, and the resulting biogeographical patterns of microbial community distribution. However, there is evidence of microbial community linkages between soil and adjacent freshwater systems^34^,^35^. Therefore, the aim of the present study was to understand the spatial distribution patterns of microbial populations and identify taxa capable of crossing the terrestrial-freshwater biome border (habitat generalists) as well as those specific to a particular habitat (habitat specialists). Moreover, we explored the co-occurrence patterns between microorganisms across the terrestrial-freshwater gradient using correlation network analysis. In this way, potential interactions among microorganisms can be dissected^20^. We analysed all three microbial domains of life, focusing on bacteria, archaea and fungi as they are all key players in ecosystem functioning. Five adjacent soils presenting different aboveground vegetation types, land managements or environmental properties were sampled around lake Erken in central Sweden, and freshwater and sediment samples were collected at different depths, including the shore line (Fig. 1). To our knowledge this is the first study to comprehensively investigate the bacterial, fungal and archaeal communities across a terrestrial-freshwater gradient.

## Results

### Microbial community distribution across the terrestrial-freshwater continuum

The different samples from the terrestrial-freshwater gradient were clustered according to the similarity of the community composition based on the main OTUs across the environmental gradient (Fig. 2). Two main clusters were evident; one including the dry hill (DH) and conifer forest (CF) site samples and the other with the rest of the sites. One sub-group within the latter was formed by the agricultural soil (AS) and flooded area (FL) samples, while the shore (SH), lake (LA) and sediment (SE) samples made up a remaining sub-group. This indicates a gradient of increasing similarity in the microbial communities from forested sites to the lake. The composition of the microbial communities within each unique site was very similar since the samples from each particular site grouped together regardless of sampling depth, which suggests habitat specificity. The exception was the samples from the dry hill (DH) and the coniferous forest (CF). These sites clustered together apart from the other sites, but differed within each of the two sites according to depth. This difference was mainly due to a lower relative abundance of *Nitrososphaerales* in the lower depths of the forest and dry hill samples, whereas the top samples displayed higher abundances (Fig. 2).

The microbial communities from the shore were more similar to the microbial communities from the lake water and sediments than to those from the other samples. In the freshwater sampled at the lake surface (LA-1 and LA-2), the most common fungal, bacterial and archaeal taxa were *Ascomycota*, *Actinobacteria* and *Thaumarchaeota*, respectively. In the deep water (LA-3), the microbial community composition was more similar to the ones observed in the sediments than to those in the littoral and surface water (LA-1 and LA-2) with lower relative abundances of *Actinobacteria* and higher relative abundance of *Proteobacteria*, respectively, as well as a higher presence of *Acidobacteria* (Fig. 2).

The distribution of the fungal taxa across the environmental gradient showed that the *Basidiomycota* (*Agaricomycetes* and *Tremellomycetes*) were dominant in the DH and CF samples, whereas the *Ascomycota* (mainly the *Dothideomycetes*, *Leotiomycetes* and *Sordariomycetes*) were more abundant in the other sites (Fig. 3). Regarding the bacterial phyla, the *Actinobacteria* and the *Proteobacteria* were present in all environmental samples, and the *Acidobacteria* in nearly all samples (Fig. 2). The *Firmicutes* and *Verrucomicrobia* were typically found in terrestrial samples, with the latter mainly at the driest sites, whereas the *Chloroflexi* were mainly detected in the deep lake samples (LA-3 and SE-Low) and the *Cyanobacteria* in the sediments from the littoral zone (SE-Top). Archaea were represented at all the sites across the environmental gradient, especially the *Thaumarchaeota* that were detected in all samples. The *Euryarchaeota*, which include methanogenic archaea (*Methanobacteriales* and *Methanosarcinales*) were predominant in the shore and sediment samples (Fig. 2).

### Ubiquity and specificity of microorganisms along the gradient

Along the environmental gradient, only a few OTUs were habitat generalists, being ubiquitously distributed all along the terrestrial-freshwater gradient, whereas the majority was more narrowly distributed and specific to a unique site or even site depth, indicating environmental niche specialization (habitat specialists). Among the habitat generalists, only six fungal OTUs belonging to the *Dothideomycetes* and the *Leotiomycetes* were detected at all sites and all depths (Fig. 3). Among these taxa, *Cladosporium* sp., *Pilidium concavum* and two *Capnodiales* were identified and they were most abundant in freshwater and mineral soils (FL-Low and SH-2; Fig. 3). Some *Tremellomycetes* affiliated to *Trichosporon* sp. and *Cryptococcus* sp. were detected in almost all samples with the exception of one freshwater sample (LA-1), and they were found in higher relative abundance in the dry hill (DH) and conifer forest (CF) soils (Fig. 3). For the bacteria, only one OTU belonging to the subgroup Gp16 of the *Acidobacteria* was widely distributed and detected in all samples but the ones from the upper layer of the dry hill (DH-Top), with higher relative abundance in the lower mineral layer of the shore site (SH-4; Fig. 3). Regarding the archaeal OTUs, we only detected *Nitrososphaerales* as habitat generalists and one OTU was present in all samples, except for the freshwater sample from the littoral zone (LA-1). The relative abundances of the *Nitrosphaerales* generalist OTUs were the lowest in the deeper layers at several sites: conifer forest soil (CF-Med and -Low), mineral layer from the shore (SH-4), sediments and freshwater (LA-3; Fig. 3).

Several microbial OTUs distributed across a wide range of different habitats were not detected in the surface freshwater sampled in the littoral zone (LA-1), and in the middle of the lake (LA-2) or within the deepest mineral layer from the shore (SH-4). This could be attributed to the lower bacterial abundances detected in these habitats (Fig. S1). These sites might potentially correspond to unique and particularly challenging habitats for bacteria. Many microbial OTUs were in fact habitat specialists, being detected at only one site and depth (Fig. 4). Within the fungal domain, the highest specificity was observed for the top mineral layer of the shore site (SH-2; Fig. 4A) with a high relative abundance of *Leotiomycetes*, *Sordariomycetes* and *Agaricomycetes*. This habitat specificity towards the SH-2 sample was not observed for bacteria nor archaea (Fig. 4B,C). Instead, habitat specialists among the bacterial OTUs were found for most of the sites, but not for the mineral soil layers of the shore (SH-2 and SH-4; Fig. 4B). In the surface lake sample (LA-2) and the deep sediments (SE-Low), high proportions of specific bacteria were observed within the *Actinobacteria*, *Bacteroidetes* and *Proteobacteria* (Fig. 4B). Except for the *Nitrososphaerales*, the deep sediment of the lake was a highly specific habitat for archaea (Fig. 4C).

### Co-occurrence patterns of microbial communities

The microbial co-occurrence patterns were explored using network inference based on strong and significant Spearman’s correlations (*ρ* > 0.06; *P* < 0.001) and using the main OTUs (relative abundance higher than 1%; Fig. 5). The resulting microbial network consisted of 710 nodes (OTUs) and 10 812 edges, representing the microbial associations, with an average degree of 15.2 node connectivity that corresponds to the number of connections to other nodes in the network. Among the total microorganisms, the fungal OTUs were the most represented within the network with 470 nodes, whereas the bacterial and archaeal OTUs were represented by 93 and 147 nodes, respectively. Five bacterial OTUs (three unclassified bacteria and two *Actinobacteria*) had the highest degree of association (degree = 54) with correlation coefficients ranging from 0.6 to 1. Within the fungal and archaeal domains, a single *Sordariomycetes* and some *Thaumarchaeota* (*Nitrososphaerales* and unclassified) had the highest degrees of association (37 and 36, respectively).

Within the bacterial domain, the *Actinobacteria* and the *Proteobacteria* were the most represented phyla with 30 and 27 nodes and an average degree of 29.9 and 17.2 node connectivity, respectively. Among the archaea, an unclassified *Thaumarchaeota* and a *Nitrososphaerales* were the most represented phyla with 53 and 52 nodes and an average degree of 15.4 and 13.9 node connectivity, respectively. Finally, in the fungal domain, the *Sordariomycetes* was the most represented phyla with 88 nodes and an average degree of 14.1 node connectivity. Some co-occurrence patterns, involving the most frequently connected microbial taxa were observed, e.g. the co-occurrence of two *Actinobacteria* (153 degrees), two *Sordariomycetes* (144 degrees), some *Nitrososphaerales* and *Sordariomycetes* (128 degrees) and between two *Agaricomycetes* (127 degrees). Focusing on the six fungal habitat generalists identified previously, only the one identified as *Pilidium concavum* did not have significant microbial association, whereas the other OTUs had degrees of association varying from 7 to 18.

## Discussion

By analysing the fungal, bacterial and archaeal community composition along a terrestrial-freshwater gradient, we identified the dominant habitat specialists and generalists within the three domains of life across the habitats along the environmental gradient. Habitat specialists were defined as OTUs detected at only one site. These OTUs could possibly be present also at other sites but at lower abundances that escape detection in the pyrosequencing assay used. However, by pooling the three sample replicates, we further increased the probability of detecting a specific OTU at a particular site even if it is rare. We thus assume that OTUs detected at only one site can be considered as habitat specialists.

Across the biomes that were sampled, different spatial patterns were observed for the different domains. As expected, members of *Actinobacteria* and *Proteobacteria* were detected along the entire gradient, which can be explained by their known diversity and dominance in the environment^36^,^37^. The *Euryarchaeota*, especially the methanogenic *Methanosarcinales*, were more abundant in the sediments and the shore samples compared to the terrestrial sites, which agrees with previous observations^13^,^38^. At the OTU level, only a few habitat generalists, but a high number of specialists were detected. Only six fungal OTUs appear to cross the biome boundaries with occurrences in all samples. Similarly, Barberán *et al*.^39^ only found 2% of all bacterial OTUs in more than 300 of the 596 soil samples they collected in Central Park. Moreover, they detected 58% of the OTUs in less than 10 of the soils analysed. The ubiquity of microorganisms can be explained by either better dispersion, higher adaptation and competition abilities or less resource needs, which all would increase their niche breadth along the terrestrial-freshwater gradient in our study^32^. Considering the significant co-occurrence patterns observed for five of the six identified fungal habitat generalists, these organisms should be both strong competitors and dispersers, whereas a trade-off between competition and colonization potentially occurs for the OTU that did not feature any significant co-occurrence pattern^40^. Slow growing oligotrophy, such as often observed for *Acidobacteria*^41^, could also be an advantage for the ubiquity of microorganisms that are more resistant to nutrient resource changes within their habitat and thus, making them able to sustain a viable population in heterogeneous environments. *Acidobacteria* were indeed widely distributed and especially subgroup 16, a common member of acidobacterial communities^42^. In the archaeal domain, only OTUs from the *Nitrososphaerales* were identified as habitat generalists being distributed along the entire gradient except in the freshwater samples from the littoral zone, corroborating previous results showing the wide range of habitats in which these ammonia-oxidizing archaea have been detected^43^. Some specific habitats did not harbour habitat generalists that were otherwise widely distributed across the sites. These habitats (the surface freshwater from the littoral zone and from the middle of the lake and the deepest mineral layer from the shore site) harbored the lowest bacterial abundances in agreement with previous work reporting lower microbial biomass in freshwater than in soil^44^. The surface freshwater from the littoral zone and from the middle of the lake along with the deepest mineral layer from the shore site may be less hospitable or more favorable to competitive non-generalist microorganisms, constituting particular niches where few habitat generalists could establish. As previously observed, niche partitioning can strongly structure microbial communities^45^. Habitat specialists are known to have a limited niche, but the highest fitness in their optimal habitat^40^. However, due to habitat specialisation, specialists are much more susceptible to changes in environmental conditions and thereby to extinction as compared to the generalists^32^. For example, even though generalist bacteria were detected in the mineral soil layer in the shore samples (*Acidobacteria*, *Actinobacteria* and *Proteobacteria*), none of these phyla and only a few unclassified bacterial OTUs were identified as habitat specialist in these samples. Moreover, except for OTUs belonging to the *Nitrososphaerales*, many archaea were specific to the sediments sampled at the deepest point of the lake. By using the ecological concept of ‘indicator species’ for archaeal lineages and analysing sequences from various environmental samples (freshwater and marine sediments and plankton, hydrothermal vents, soils), Auguet *et al*.^13^ concluded that the *Methanomicrobiales* could be considered as an indicator group of freshwater sediments. In our study, the *Methanomicrobiales* did not constitute the most important specialist archaeal taxa in the sediments. Instead, the sediments were a more specific habitat for *Thaumarchaeota* and *Thermoplasmatales*, indicating that the concept of ‘indicator species’ is context dependent and therefore difficult to apply globally.

Interestingly, the upper mineral soil layer from the shore harboured a high proportion of habitat-specific fungi. Whereas the relative abundance of the habitat specialists *Leotiomycetes* and *Sordariomycetes* coincided with overall fungal abundances across the sites this was not the case for the habitat specialist *Agaricomycetes*, which were relatively more abundant locally than the total *Agaricomycetes* community. This indicates that the habitat specialist distribution across an environmental gradient does not necessarily reflect the one at the metacommunity level and that specialist taxa are not always rare taxa as previously hypothesized^33^. Habitat specialist could be considered as species banks for dispersal and colonization of new and favorable habitats facilitated by production of resting cysts or spores which can survive long periods and disperse through air and water^46^,^47^. In the present study, the shore site, through its physical localisation between the soil, lake water and sediments, could represent a transitional dispersion area for microbial populations between these different environments. This site is subjected to flooding events which would allow inoculation of the lake by soil microorganisms and vice versa due to the physical exchange, as proposed by Crump *et al*.^35^. Vertical dispersal could also explain the similarity observed between the deep lake water and the sediments, which both harboured similar relative abundances of *Actinobacteria*, *Proteobacteria*, *Acidobacteria* and *Chloroflexi*. The exchange might be driven by both sedimentation in the water column and by the nutrient fluxes at the water-sediment interface^48^.

Contrasting community assembly mechanisms have been proposed for habitat generalists and specialists, either through species sorting or spatial factors^32^,^33^. However, considering that these two groups would respond differently to environmental and spatial factors, the spatial turnover of microbial communities will be modified according to their relative importance. In the present study, we observed that microbial communities were more similar within habitats than across habitats, most likely due to environmental filtering as suggested by the few habitat generalists detected. Species sorting could also result from biotic interactions between microorganisms such as competition, mutualistic or syntrophic relationships. These interactions were analysed and visualized through the co-occurrence analyses and association network, which may correspond to microorganisms performing similar or complementary functions and/or sharing similar preferred environmental conditions, but not necessarily having physical interactions^27^,^49^,^50^. Interestingly, while the fungal taxa were the most represented within the network, bacteria, especially two actinobacterial OTUs, had the highest number of associations. This suggests that they have a large impact on the microbial community structure along the environmental gradient. The network was supported by both microbial intra-and inter-phyla associations and underlined the importance of *Actinobacteria*, *Sordariomycetes*, *Agaricomycetes* and *Nitrososphaerales* in the complex ecological interactions shaping the microbial community structure across the biomes studied.

## Conclusion

By analysing bacterial, fungal and archaeal community composition across a terrestrial-freshwater gradient, we identified only a few microbial OTUs as habitat generalists able to cross biome boundaries. Many habitat specialists were detected at each site, which suggests that environmental filtering was an important assembly mechanism for microorganisms within all three domains and that dispersal was limited. This low ubiquity of microorganisms shows the susceptibility of microbial communities to changing environments. However, areas allowing dispersal, such as the shore that links soil and freshwater, were likely important to maintain diversity across the environmental gradient.

## Materials and Methods

### Environmental samples

**Figure 1:**
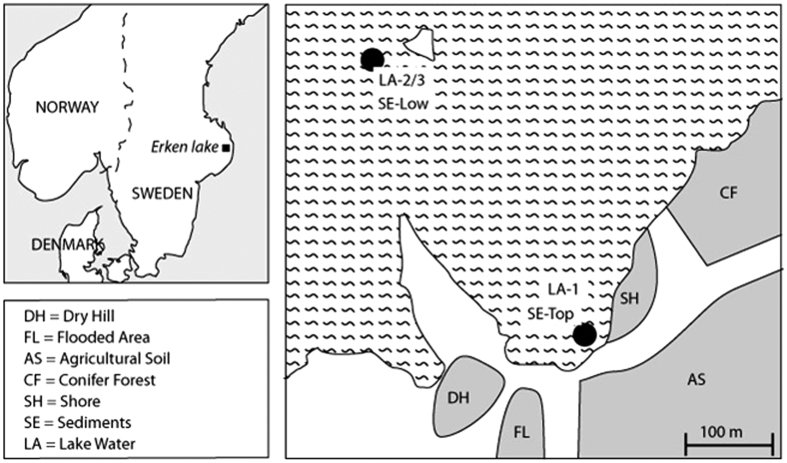
Location and description of sampling sites. The figure was composed using Adobe Illustrator CS3 software (www.adobe.com) based on Google Earth (Map data: Google, DigitalGlobe).

**Figure 2:**
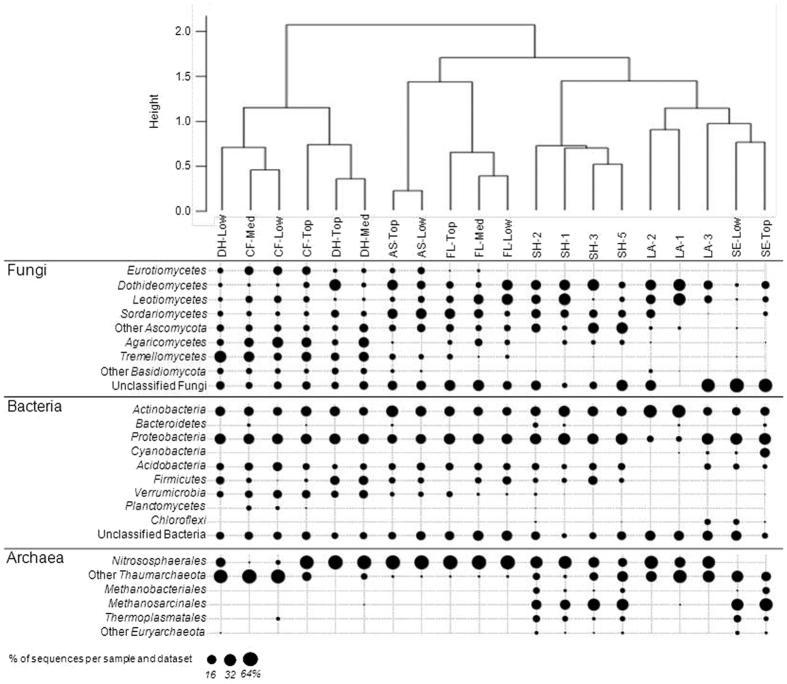
Similarity of community composition as revealed by hierarchical clustering of the distribution of the main OTUs with a dot plot indicating the relative abundance of the main microbial taxa at different depths in the environmental samples: Dry hill (DH), Conifer forest (CF), Flooded area (FL), Shore (SH), Agricultural soil (AS), lake side (LA-1), Lake surface (LA-2), 10 m depth lake (LA-3), Sediments at the lake side (SE-Top) and in the middle of the lake (SE-Low). Each sample corresponds to the averages of the biological triplicates (Fig. S2).

**Figure 3:**
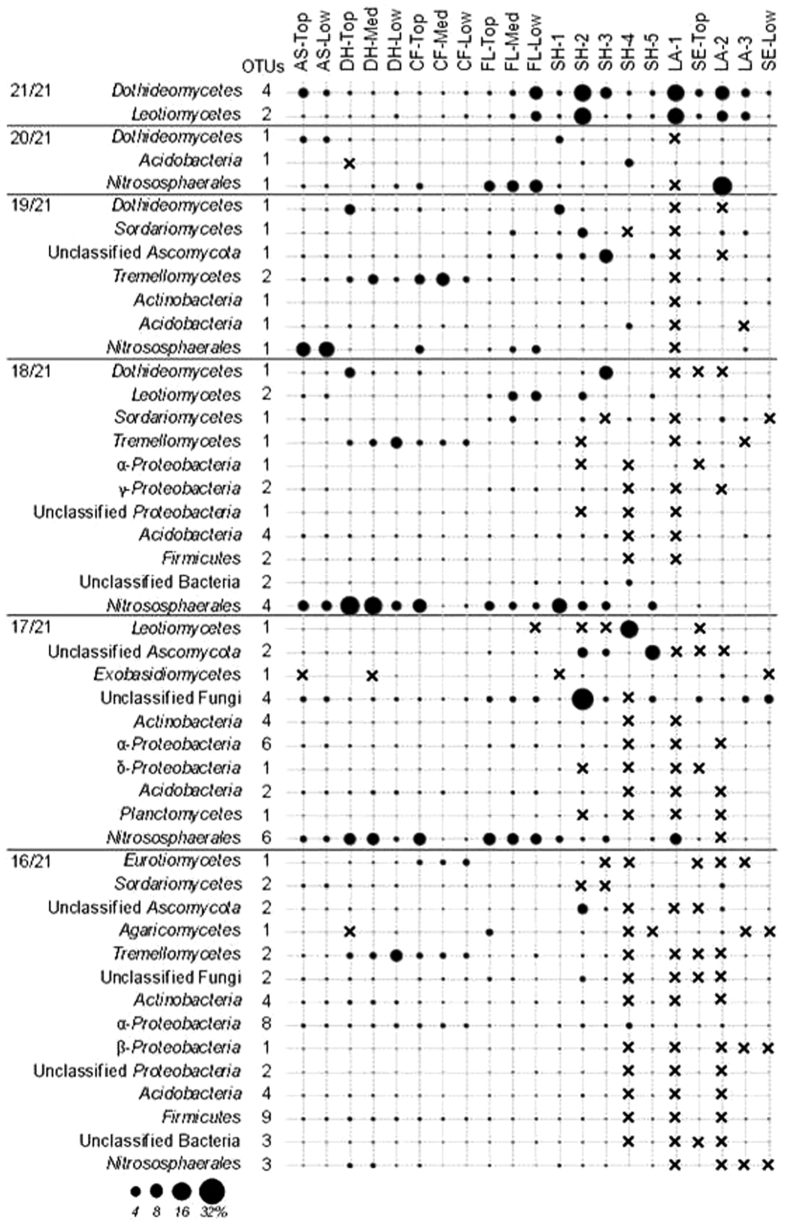
Dot plot of the relative abundance of the habitat generalists detected in all (occurrence 21/21) and almost all the sampling sites (occurrence <21/21). Each sample corresponds to the averages of the biological triplicates. Crosses indicate no detection.

**Figure 4:**
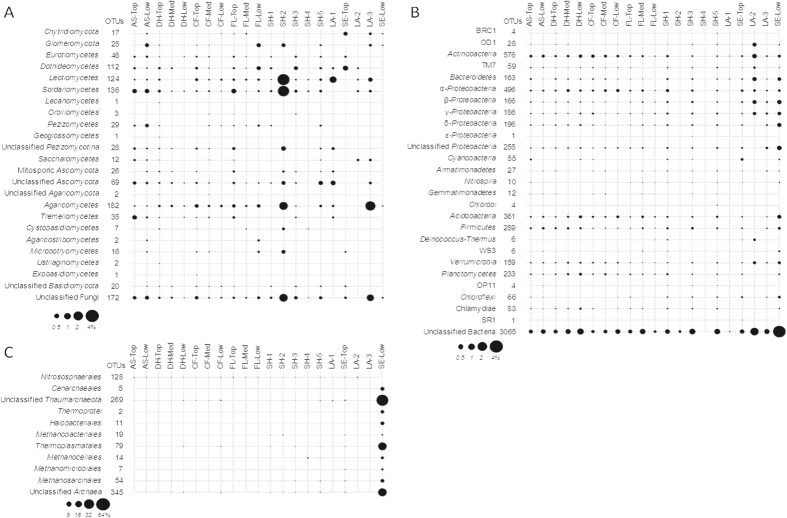
Dot plot of the relative abundance of the habitat specialists (OTUs within microbial taxa) detected in only one site and depth along the terrestrial – freshwater biomes taxa ((**A)** Fungi; (**B)** Bacteria; (**C**) Archaea).

**Figure 5:**
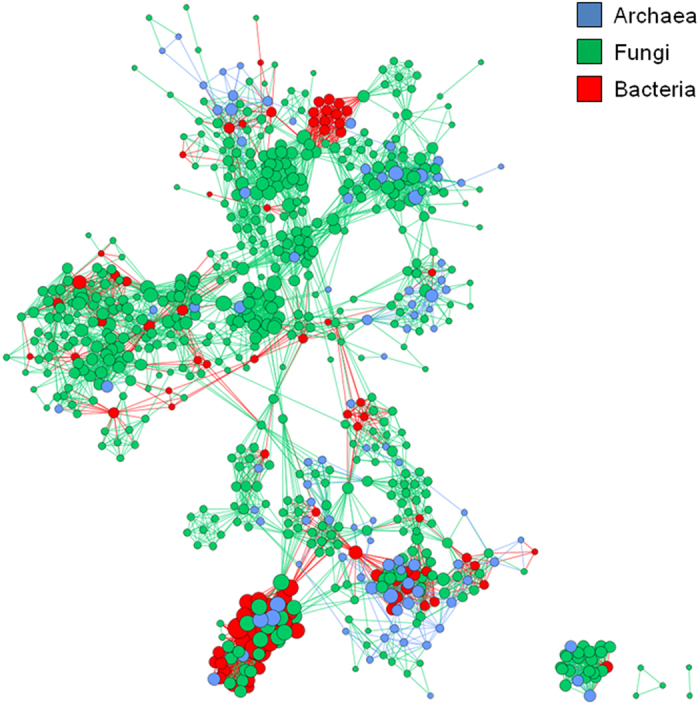
Co-occurrence network of the main OTUs based on correlation analysis. Nodes correspond to microbial OTUs and edges to the microbial associations. A connection stands for a strong (Spearman’s ρ > 0.6) and highly significant (P < 0.001) correlation. The size of each node is proportional to the number of degree.

To create a terrestrial-freshwater gradient, soil, sediment and water were sampled in adjacent sites and within lake Erken in central Sweden (59°51′N, 18°36′E) in October 2009 (Fig. 1). Soil cores (22 cm depth × 3 cm diameter) were sampled at five different sites differing in their vegetation, characterised and denoted as ‘Dry hill’ (DH) dominated by *Quercus robur* in the tree layer, ‘Flooded area’ (FL) dominated by *Alnus glutinosa* in the tree layer, ‘Agricultural soil’ (AS) dominated by annual cereal crops, ‘Conifer forest’ (CF) dominated by *Picea abies* in the tree layer, and ‘Shore’ (SH) mainly covered by *Salix* in the bush layer. For each site, three replicates were sampled. According to the soil profile, each soil core was subsampled either into i) a top (0–5 cm depth), a medium (8–13 cm depth) and a lower (17–22 cm depth) layer (DH, CF and FL -Top/-Med/-Low) or ii) a top (0–5 cm depth) and a lower (17–22 cm depth) layer (AS -Top/-Low). The FL-Low layer was visually a clay-rich mineral soil. The SH cores were divided into five equal layers since they were composed of alternated organic (SH -1/-3/-5) and mineral-sandy layers (SH-2/-4). Each subsample was homogenised by sieving (2 mm mesh size).

Lakewater samples were obtained from the littoral zone (LA-1), and from both surface (1 m depth; LA-2) and 10 m depth (LA-3) at the deepest point of the lake. Three replicates of 500 mL of water were immediately filtered through 0.22 μm pore size polycarbonate filters. Sediment samples (50 mL) were collected from the littoral zone (SE-Top) and from the central part of the lake (21 m depth; SE-Low) in three replicates from each site. All samples (soil cores, filters and sediments) were stored at −20 °C for subsequent molecular analysis.

### DNA extraction

DNA from soil and pelletized sediments was extracted from 4 × 0.5 g aliquots. DNA from water samples was obtained by dividing the filters into four pieces and extracting the DNA from each filter piece individually in order to improve the extraction yield. The Griffiths protocol^51^ was used with the modifications described by Monard *et al*.^52^. DNA quality and quantity were checked at 260 nm (NanoDrop Technologies). All 4 extraction-replicates were pooled and stored at −70 °C.

### PCR amplification and pyrosequencing

The bacterial 16 S rRNA gene amplifications were performed using the 341 F and 805 R primers^53^ and the fungal ITS amplifications using the ITS1F^54^ and ITS4^55^ primers. For the Archaea, nested PCR amplifications of the 16 S rRNA gene were performed because some samples were not successfully amplified with the direct archaeal-specific arch344f^56^-806R^57^ primer set. The arch21F-958R primer set^58^ was used for the first round of amplifications, followed by the arch344f-806R primer set for the second round of amplifications. The 805 R, ITS1F, ITS4 and arch806R primers contained a unique additional 6 bp length barcode used to tag each PCR product according to the original environmental sample and the bacterial and archaeal primer sets were supplemented with the 454 FLX adaptors ‘A’ and ‘B’.

All PCR amplifications were performed in duplicates using 4 μL of DNA diluted 100 and 1000 times as template. The PCR reaction further contained 2.5 units of DreamTaq green DNA polymerase (Fermentas), 1 X PCR buffer supplied by the manufacturer, 1.6 mM MgCl_2_, 80 μM of dNTP, 1.6 μg of BSA, 0.4 μM of each primer and H_2_O to a final volume of 40 μL. The PCR conditions were: 94 °C for 5 min, followed by between 26 and 31 cycles of denaturation (94 °C; 30 sec), annealing (53, 55 and 50 °C for the bacteria, fungi and archaea, respectively; 40 sec) and extension (72 °C; 30 sec); followed by the final elongation (72 °C; 7 min). The number of PCR cycles was determined according to previous qPCRs performed on our DNA samples (Fig. S1). All diluted DNA extracts were amplified in duplicates.

Each PCR sample was purified using the GeneJet purification kit (Fermentas) following the manufacturer’s instructions and quantified using a Qubit Fluorometer (Invitrogen) and an equal amount of DNA (25 ng) from each sample and each DNA dilution was pooled. To remove potential primer dimers, the pooled DNA was finally gel purified using the Qiaquick gel extraction kit (Qiagen). The final samples were sent to LGC Genomics and ligation of the 454 FLX sequencing adaptors ‘A’ and ‘B’ was performed on the fungal amplicons. The bacterial, fungal and archaeal amplicons were then sequenced on a 454 Genome Sequencer FLX (Roche) machine from the 805 R, both ITS1F and ITS4 and the arch806R sides, respectively. The fasta and quality files from each sample have been submitted to the Sequence Read Archive (www.ncbi.nlm.nih.gov/sra; accession SRX981059).

### Processing of pyrosequencing data

The two fungal sequence datasets (ITS1 and ITS2) were processed using the SCATA pipeline (http://scata.mykopat.slu.se refs 52,59) whereas the pipeline provided by the Ribosomal Database Project^60^ was used for both bacterial and archaeal sequences. Adaptor and primer sequences were trimmed, and sequences of low quality (exponential quality score lower than 10) or shorter than 200 bp were removed. The 6 bp DNA barcode attached to the primers was used to assign sequences to samples. Operational taxonomic units (OTUs) were defined using single linkage clustering at the 98.5% identity level for the fungal sequences according to Wallander *et al*.^61^ and Blaalid *et al*.^62^. For bacterial and archaeal sequences, OTUs were defined using complete linkage clustering at the 97% identity level^63^. After removing all the singletons, a total of 641 230, 182 365 and 624 321 sequences were obtained for the bacterial, fungal and archaeal communities, respectively. These corresponded to 41 418, 2 295, 2 062 and 3 497 bacterial, fungal (ITS1 and ITS2) and archaeal OTUs respectively, distributed across the 21 environmental samples replicated three times. The OTUs were taxonomically identified using the RDPII taxonomy tool^63^ and the SILVA-ARB database^64^ for the bacterial and archaeal OTUs, respectively (see Table S1 for details). The fungal ITS sequences were phylogenetically assigned using MEGAN v.4. (MEtaGenome Analyzer, Center for bioinformatics, Tübingen, Germany^65^) according to their best matches from the GenBank database^66^.

### Community and statistical analysis

After checking that the Bray-Curtis distance within the three replicates were significantly lower than between depths or sites (vegdist function in the Vegan library program implemented in R (http://www.r-project.org/); *P* < 0.001, *t*-test; Fig. S2), data from the different sample replicates were pooled to increase the amount of sequences and the representativeness of each sample. The pooled data were then clustered according to the dissimilarity (Bray-Curtis) of their microbial community composition. The main OTUs (relative abundance per sample higher than 1‰ for each dataset – fungi, bacteria and archaea) in the different environmental samples, the habitat generalists detected in at least 16 of the 21 samples and the habitat specialists detected in only one sample type were visualised using the Perl script bubble.pl (available at http://hallam.microbiology.ubc.ca/).

To explore co-occurrence patterns, a network analysis was performed in R by calculating all possible Spearman’s rank correlations between OTUs with a relative abundance per sample higher than 1%. This filtering step removed poorly represented OTUs and reduced network complexity. A valid co-occurrence event was considered to be a robust correlation if the Spearman’s correlation coefficient (ρ) was both >0.6 and highly statistically significant (*P* < 0.001). Global descriptors of the network were obtained using Network Analyzer in Cytoscape v3.0.1^67^ and the network of coexisting microorganisms was visualised using the Gephi v0.8.2-beta software^68^.

## Additional Information

**How to cite this article**: Monard, C. *et al*. Habitat generalists and specialists in microbial communities across a terrestrial-freshwater gradient. *Sci. Rep*. **6**, 37719; doi: 10.1038/srep37719 (2016).

**Publisher's note:** Springer Nature remains neutral with regard to jurisdictional claims in published maps and institutional affiliations.

## Supplementary Material

Supplementary Information
