# Supplementary material for: Habitat generalists and specialists in microbial communities across a terrestrial-freshwater gradient
Source: Sci Rep. 2016 Nov 25;6:37719. doi: 10.1038/srep37719 (PMC5123577; doi:10.1038/srep37719)

Habitat generalists and specialists in microbial communities across a terrestrial-freshwater gradient

MONARD C.[1a], GANTNER S.[1,2b], BERTILSSON S.[2], HALLIN S.[1], STENLID J.[1]

Table S1. Details of the amount of reads and OTUs obtained from the 454 barcoded pyrosequencing analysis for the four datasets (A) and for each dataset (B).

A

Dataset	Total reads	Good reads	Singletons	OTUs
ITS1	719 587	101 115	4 734	2 295
ITS2	719 587	81 250	4 064	2 062
Bacteria	743 441	641 230	16 180	41 418
Archaea	856 453	624 321	1 457	3 497

B

		Fungi			Bacteria			Archaea		
		Good reads	Singleton	OTUs	Good reads	Singleton	OTUs	Good reads	Singleton	OTUs
ITS1	AS-Top	1 296	162	369	39 708	900	10 494	40 889	68	1 936
	AS-Low	3 465	265	434	33 464	1 065	10 098	42 002	60	1 782
	DH-Top	11 696	522	790	66 075	1 243	14 086	36 726	16	506
	DH-Med	5 830	239	487	41 586	1 354	10 622	36 461	18	602
	DH-Low	1 860	68	246	38 472	563	8 122	36 685	24	425
	CF-Top	14 323	606	978	42 485	640	9 392	31 071	44	916
	CF-Med	14 325	360	756	39 312	720	7 524	31 090	24	386
	CF-Low	7 322	180	475	29 449	922	6 515	36 714	52	713
	FL-Top	13 662	742	1 055	54 113	1 045	13 014	52 546	82	1 997
	FL-Med	4 694	210	441	30 415	1 654	8 790	26 011	28	1 611
	FL-Low	3 618	101	186	12 960	220	3 214	16 589	32	898
	SH-1	4 239	288	350	47 156	1 304	12 022	37 583	60	1 145
	SH-2	242	14	33	492	7	356	31 461	31	616
	SH-3	1 740	75	116	31 621	910	8 405	39 813	63	920
	SH-4	4 114	45	65	23	0	17	9 068	5	97
	SH-5	3 828	325	474	52 167	1 159	12 233	28 707	121	1 538
	SE-Top	1 077	166	239	27 579	965	5 872	36 071	153	1 303
	SE-Low	775	164	189	38 798	1 214	7 989	32 958	548	1 785
	LA-1	757	40	55	1 651	27	203	7 864	15	118
	LA-2	314	26	50	6 608	48	434	12 685	10	79
	LA-3	1 938	136	206	7 096	220	2 005	1 327	3	51
ITS2	AS-Top	840	115	259						
	AS-Low	2 276	213	353						
	DH-Top	12 265	552	858						
	DH-Med	3 549	150	419						
	DH-Low	1 563	48	214						
	CF-Top	12 324	545	815						
	CF-Med	9 447	219	590						
	CF-Low	4 013	119	355						
	FL-Top	10 355	658	975						
	FL-Med	3 618	183	395						
	FL-Low	4 120	114	168						
	SH-1	4 114	295	341						
	SH-2	187	12	34						
	SH-3	2 586	86	149						
	SH-4	3 476	30	52						
	SH-5	1 895	195	261						
	SE-Top	301	91	123						
	SE-Low	995	232	263						
	LA-1	1 230	39	70						
	LA-2	201	19	36						
	LA-3	1 895	149	207						

Fig S1. Fungal (ITS gene copy number; A), bacterial (16S gene copy number; B) and archaeal (16S gene copy number; C) abundances in the soil (per gram of dry soil), sediments (per gram of dry sediment) and water (per liter) samples determined by qPCR.

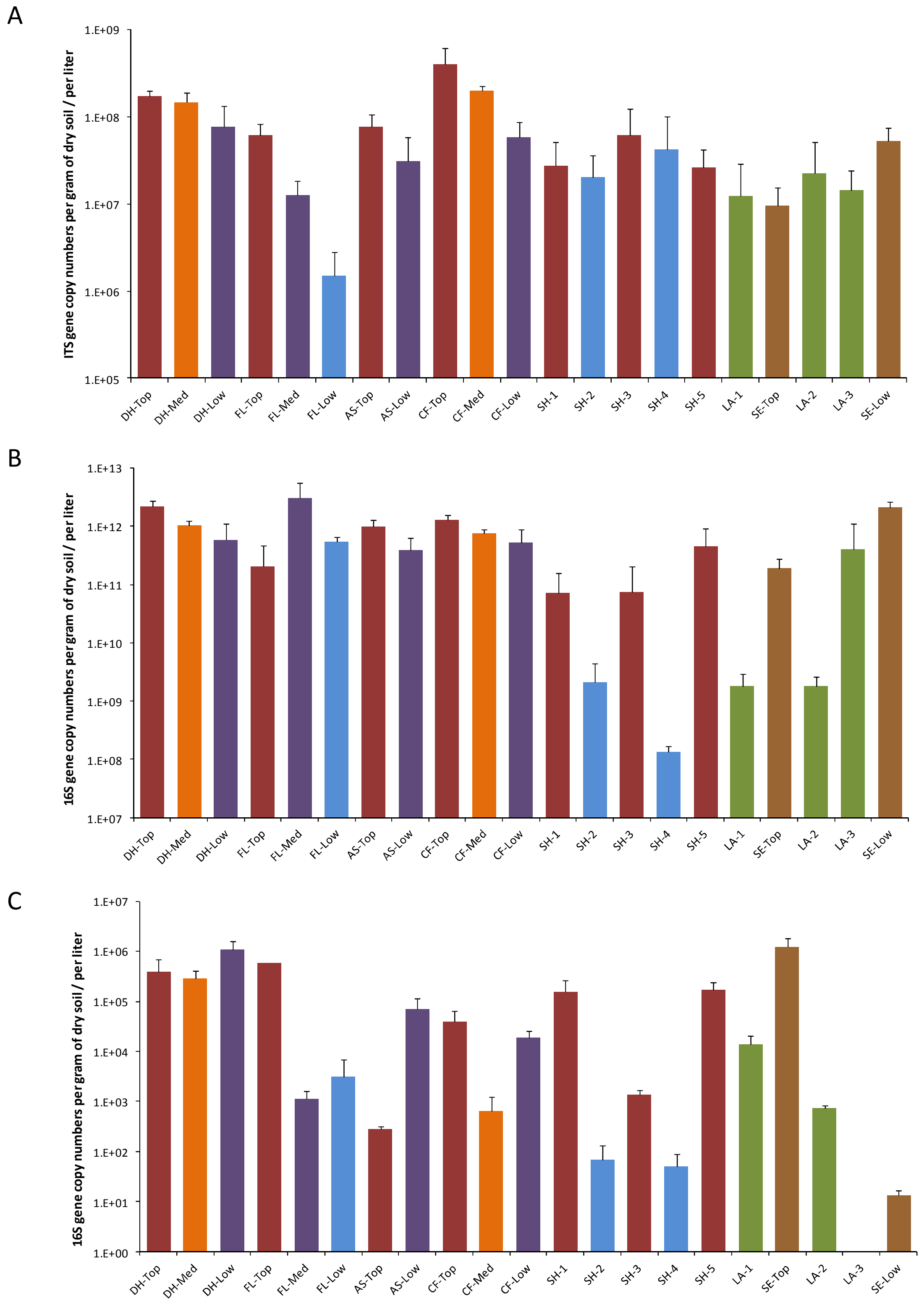

Fig. S2. Dendrogram presenting the Bray-Curtis dissimilarity of the microbial composition (main OTUs) in the three replicated samples across environmental samples.

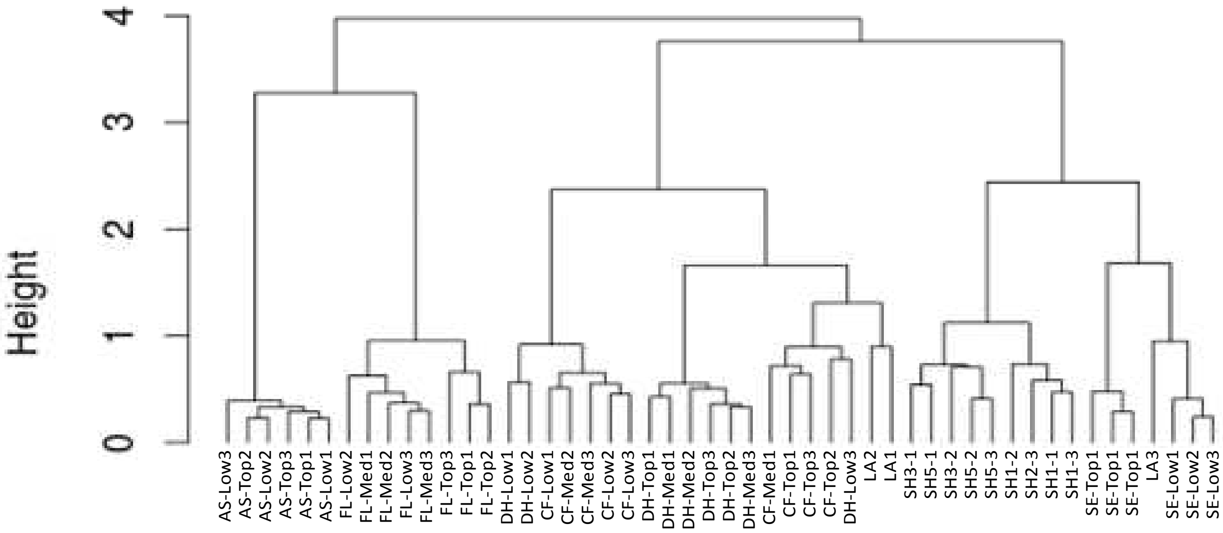

Supplement: Supplementary Information [file srep37719-s1.pdf]
